# Single‐stage hepaticojejunostomy for symptomatic portal biliopathy in a splenectomized patient: A report of two cases

**DOI:** 10.1002/ccr3.3013

**Published:** 2020-06-01

**Authors:** Narendra Pandit, Laligen Awale, Lokesh Shekher Jaiswal, Shailesh Adhikary

**Affiliations:** ^1^ Department of Surgery B P Koirala Institute of Health Sciences (BPKIHS) Dharan Nepal

**Keywords:** hepaticojejunostomy, portal biliopathy, shunt surgery

## Abstract

Single‐stage biliary decompression without prior shunt surgery, although risks for catastrophic bleeding, it is feasible to perform upfront hepaticojejunostomy in a splenectomized patient where shuntable vein is not available in patient with portal biliopathy.

## Graphical abstract

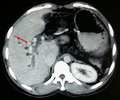

## 1 INTRODUCTION

Portal biliopathy (PB), an uncommon cause of benign biliary obstruction, refers to the biliary ductal and gallbladder wall abnormalities in patients with portal hypertension. It usually occurs secondary to cavernomatous transformation of the portal vein. These changes are seen in decreasing order of frequency in patients with extrahepatic portal vein obstruction (EHPVO), and cirrhosis and noncirrhotic portal hypertension (NCPF).^1^ It takes the form of biliary radicle dilatation, irregularities, stricture, and stone formation. The changes are caused by compressive and ischemic effects of portal cavernoma in the biliary region.^2^ Approximately 20% of these patient presents with biliary symptoms (pain, pruritus, jaundice, and cholangitis).^3^ Treatment of PB could be done endoscopically (sphincterotomy, stone extraction, or biliary stenting of the common bile duct) alone or as a bridge to surgery and can act as definitive procedure. However, the definitive surgery includes decompressive portosystemic shunt followed by bilioenteric anastomosis, if necessary.^1^, ^3^, ^4^

Upfront biliary drainage is often associated with catastrophic bleeding and death from the abundant venous collaterals around the bile duct. Furthermore, the success of the anastomosis is limited by collaterals around the bile duct.^5^ Unfortunately, prior shunt surgery is often not possible in some subsets of patients because of the extent of thrombosis or the lack of shuntable vein especially in splenectomized patient. In this setting, biliary bypass without portal decompression becomes an important management option, despite the risk of bleeding.^3^, ^5^, ^6^ Here, we describe a successful single‐stage hepaticojejunostomy in two patients with symptomatic portal biliopathy postsplenectomy, where the shuntable vein was not available for prior portal decompression.

## 2 CASE 1

A 20‐year‐old female patient presented to us (hepato‐pancreato‐biliary surgery team) with two months history of epigastric pain and intermittent fever. She denied any history of recent hematemesis and bleeding from nose and gum. She underwent open cholecystectomy, common bile duct (CBD) exploration and T‐tube placement for cholelithiasis and obstructive jaundice due to the CBD stone four months back in our hospital by the general surgery team. Intraoperative and postoperative periods were uneventful, and the T‐tube was removed at six weeks of surgery without any sequelae. On further enquiring, her past history was significant for hematemesis at age of eight years, requiring emergency splenectomy by thoracoabdominal approach suggesting massive splenomegaly with portal hypertension bleed. Since then, she had been asymptomatic for the last 12 years.

**FIGURE 1:**
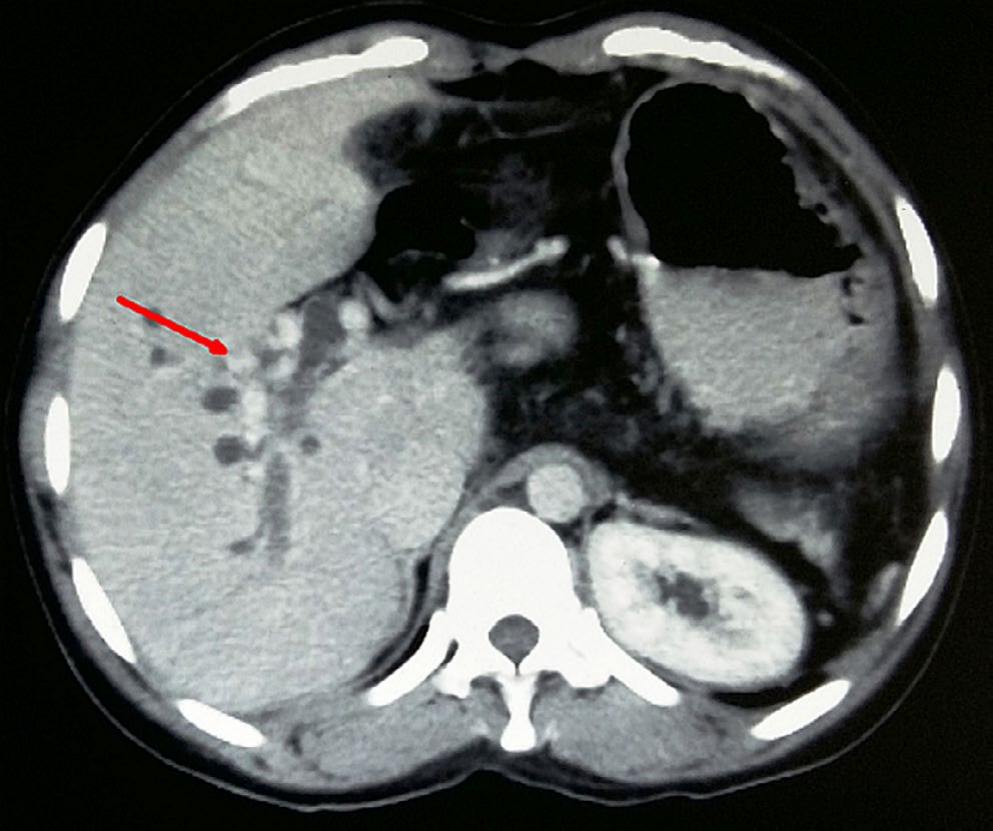
Contrast‐enhanced CT abdomen showing cavernomatous transformation (arrow) of main portal vein with intrahepatic bile duct dilatation and absence of spleen on the left side

**FIGURE 2:**
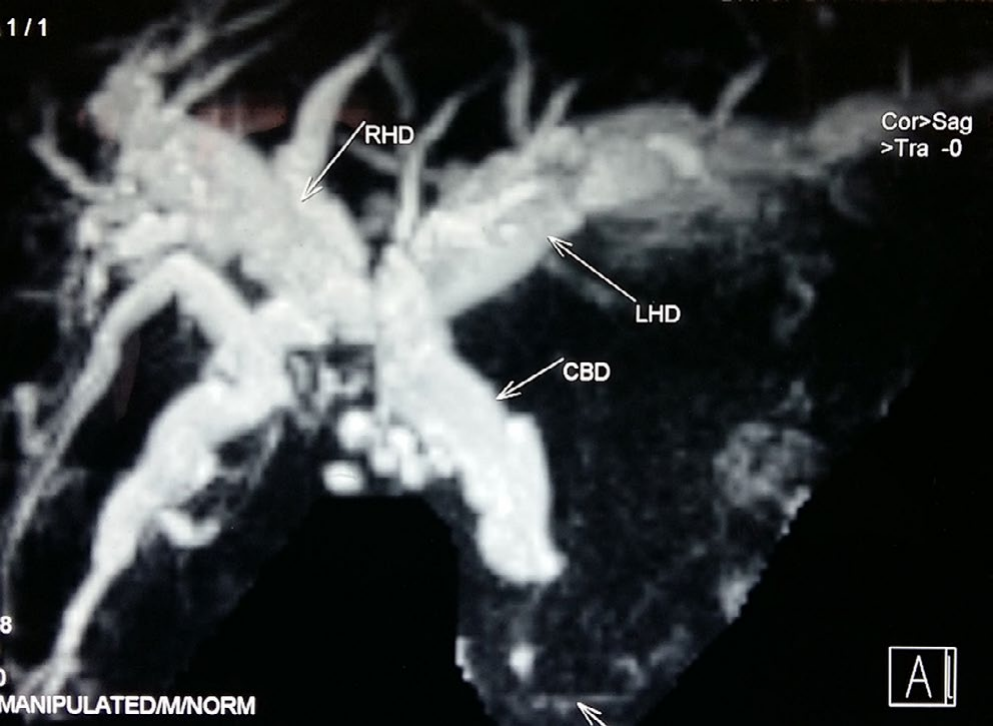
T2 Magnetic resonance imaging showing long segment distal common bile duct stricture with proximal biliary dilatation due to the portal biliopathy

On general examination, she was thin‐built, vitals stable without jaundice. Abdominal examination was normal, apart from the scar of previous two surgeries. Laboratory investigation showed normal hemoglobin, total leukocyte, and platelet counts. Her coagulation profile (prothrombin time, activated partial thromboplastin time, and fibrinogen) too was normal. Her liver function test was significant for raised alkaline phosphatase level, which was two times the upper limit of normal with a normal range of bilirubin level. An ultrasonography of abdomen with Doppler study revealed main portal vein being replaced by multiple prominent collateral vessels at the portal hepatis with bilateral intrahepatic biliary radicle dilatation. The liver echotexture appeared normal. On upper gastrointestinal (GI) endoscopy, there were low‐grade esophageal varices. In view of past surgery for stone disease, portal collaterals, and requirement of splenectomy for probable variceal bleed at young age, a diagnosis of portal biliopathy with EHPVO was made and underwent magnetic resonance cholangiopancreatography (MRCP) and contrast‐enhanced computer tomographic (CT) scan of the abdomen. It revealed long segment, smooth lower bile duct stricture with proximal biliary dilatation (Figure 1). Moreover, there were multiple collaterals at the hilum (Figure 2) with nonvisualization of main portal, splenic, and proximal superior mesenteric vein.

As no shuntable vein was available for prior portal decompression and looking at the safe landing of prior cholecystectomy and CBD exploration, single‐stage hepaticojejunostomy was planned. Intraoperatively, there were multiple collaterals (average diameter‐ 2 mm) at pericholedochal region, which were ligated. The dilated proximal bile duct was identified by needle aspiration of bile, and a small window was made on it of approximately 2 cm size. A Roux‐en‐Y hepaticojejunostomy (side‐to‐side) was done. Intraoperatively, there was 250 mL of blood loss with operative time of 180 minutes. Postoperative period was uneventful, and she was discharged on 6th postoperative day. Liver biopsy revealed well‐preserved architecture pattern with mild periportal fibrosis suggesting noncirrhotic portal hypertension. At 38 months of follow‐up, abdominal ultrasonography and liver function test were normal. Moreover, she had one episode of well‐tolerated esophageal variceal bleed requiring two sessions of banding.

## 3 CASE 2

A 30‐year‐old male patient presented with seven days history of right upper quadrant abdominal pain, low‐grade fever, and progressive jaundice. His past history was significant for symptomatic hypersplenism without upper GI bleed for idiopathic noncirrhotic portal fibrosis for the last 4 years. During this period, he required multiple hospital admission and packed red blood cell transfusion. He also had early satiety and feeling of heaviness in the left upper quadrant of the abdomen due to the massive splenomegaly. He denied any history of alcohol consumption. Upper GI endoscopy revealed low‐grade esophageal varices. For this, he underwent splenectomy six months back at our center. At first admission, his coagulation work‐up were within normal limits. Moreover, liver biopsy confirmed noncirrhotic portal hypertension changes. Decompressive shunt surgery was not done during splenectomy as the low‐grade esophageal varices were already decompressed by the presence of spontaneous large splenorenal shunt as observed by the presence of dilated left renal vein. Moreover, the patient did not have hematemesis due to the gastric or esophageal varices.

On examination, he was thin‐built, febrile, with scleral icterus. His blood pressure and pulse rate were within normal limit. Abdominal examination revealed palpable and nontender gallbladder. Laboratory findings showed leukocytosis (13 400 cells/mm^3^), with normal serum biochemistry and renal function test. His liver function test was deranged with raised bilirubin and alkaline phosphatase levels (total bilirubin: 14 mg/dL; direct bilirubin: 10 mg/dL; alkaline phosphatase: 256 U/L). An abdominal ultrasonography with Doppler study revealed increased liver echogenicity with biliary radicle dilatation extending to the distal common bile duct. The gallbladder was distended without stones in the biliary tract. Surprisingly, the portal vein was replaced by the portal cavernoma, with nonvisualization of spleno‐mesentero portal axis, which was not seen at the time of previous surgery. Contrast‐enhanced CT abdomen showed multiple collaterals at the porta hepatis compressing the distal bile duct. A diagnosis of portal cavernomatous biliopathy with cholangitis was made, and he was commenced on empirical broad‐spectrum intravenous antibiotic. Patient responded well in the next 5 days with absence of fever and normalization of leukocyte counts and planned for single‐stage biliary decompression. Shunt procedure including splenorenal, portocaval, or mesocaval shunt was not feasible.

Intraoperatively, gallbladder and the CBD were dilated till the lower end. There were multiple, large (average diameter 2 mm) pericholecystic and pericholedochal collaterals. Cholecystectomy was performed using retrograde approach. The dilated proximal bile duct was identified by tracing the cystic duct. A window of 2 cm was created on the dilated portion, and a Baker's probe was placed inside the lumen to check for any calculi, which led to torrential bleed from the intracholedochal varices. It was controlled with Pringle's maneuver coupled with controlled oversewing of the distal bile duct. Side‐to‐side Roux‐en‐Y hepaticojejunostomy was performed. The intraoperative blood loss was 700 mL, and the operating time was 190 minutes. Two units of packed red blood cells were transfused. Postoperatively, serum bilirubin level dropped gradually and came to the normal range at 10th day. Subsequently, he developed ascites which was managed with diuretics. At 28 months of follow‐up, patient is asymptomatic with normal liver function test.

## 4 DISCUSSION

There is no consensus regarding optimal management of portal biliopathy in splenectomized patient, as the data regarding various forms of therapy are based on the short series and personal experience. Hence, the treatment of symptomatic patients should be determined on individual case approach.^7^

Endoscopic retrograde cholangiopancreatography (ERCP) with biliary decompression is the first line of management in symptomatic patient. In the presence of strictures, stones, and cholangitis, symptoms can be relieved by sphincterotomy, stone extraction. and stenting, but at the risk of bleeding from the choledochal varices.^1^, ^3^, ^8^ Hence, the procedure should be done meticulously and by an experienced team. Moreover, the endoscopic treatment is only temporary, and it acts as a bridge to surgery in the presence of dominant stricture where the stents has to be exchanged frequently due to the recurrent blockage.^9^, ^10^ Also, it is not the treatment of choice for the patients hailing from the remote areas with limited access to endoscopic treatment.^7^

Surgery is the best and one time treatment modality especially in young patients from far off places with severe biliary abnormalities, persistent symptoms, or dominant stricture.^1^ They usually require initial decompressive shunt surgery (proximal splenorenal or mesocaval shunt). If symptoms of biliary obstruction persist, a biliary diversion should be considered as a second surgical procedure. In approximately 50% of cases, the initial shunt surgery decompresses the hilar collaterals, relieving the biliary obstruction in six weeks to three months. The remaining 50% of patient requires second stage hepaticojejunostomy.^4^, ^11^ The second stage surgery becomes relatively safer as the collaterals at hilum are decompresssed, reduced in number, and at low pressure leading to lesser intraoperative bleeding while approaching the bile duct.^3^, ^5^ In selected patients with hepatolithiasis, where initial ERCP is not technically possible, simultaneous shunt and hepaticojejunostomy can be safely performed using the Pringle maneuver to control any bleeding.^12^ In the present cases, neither splenic vein nor the superior mesenteric vein were available due to the thrombosis of splenoportal and mesentericoportal axis for Linton or mesocaval shunt.

Upfront or single‐stage Roux‐en‐Y hepaticojejunostomy has been reported in the literature for symptomatic biliary obstruction, although seems to be too risky due to the massive bleeding and on‐table death.^6^, ^13^ A study by Cellich et al, from Sydney, Australia, was done, where three out of eight cases underwent single‐stage biliary bypass, and showed impressive results without significant bleeding and mortality. The procedure rendered all three patients asymptomatic, concluding that biliary decompression without prior shunt surgery can be performed successfully, if necessary.^5^ Similarly, a study from India by Agrawal et al also showed excellent results of biliary bypass without prior shunt surgery in patients with postcholecystectomy biliary stricture with portal hypertension.^13^ However, single‐stage biliary bypass carries the risk of increased intraoperative blood loss and operative time. Furthermore, the surgeons should be ready to abandon the procedure, should there be a massive bleeding due to opening of collaterals as concluded from the study by Perakath et al, from South India.^6^

Hence, the success of single‐stage procedure depends on meticulous dissection and ligating all the collaterals around the dilated portion of the bile duct. The aim should be to make a "window" in the bile duct, just below the confluence for side‐to‐side anastomosis (instead of end‐to‐side), rather than dissecting the whole length of biliary tree (to avoid damaging the collaterals of portal system) as was preferred in our patients. Moreover, there should be liberal use of harmonics, Pringles maneuver, appropriate selection of patients, and commencing vasoactive agents intraoperatively (somatostatin, octreotide, or terlipression) to reduce portal pressure.^7^ Above all, the most important factor for the success is the surgeon's patience. We attempted single‐stage biliary bypass in our patients with the expectation of reduced number and pressure of collaterals at the hilum due to the prior splenectomy, which usually decompresses the portal pressure to some extent. Moreover, both the patients were thin‐built (facilitating the surgery), and we had no alternative in the first patient as there was no shuntable vein (splenorenal or mesocaval) and the second patient developed symptoms of portable biliopathy despite large spontaneous splenorenal shunt.

Shunt surgery significantly decreases the portal pressure and addresses the variceal bleed and biliopathy. Moreover, nonshunt surgery like splenectomy decreases the portal blood flow, thus reducing the pressure at pericholedochal collaterals. This sometimes acts as a remedy for biliopathy.^14^ But our second patient paradoxically developed biliopathy due to the diffuse portal and splenic vein thrombosis resulting as a complication of splenectomy. We could successfully perform cholecystectomy and bilioenteric anastomosis by a retrograde approach, which helped us to identify the dilated portion of the bile duct. The collaterals overlying the bile duct were over sewn with the liberal application of Pringle's maneuver, and the bilioenteric anastomosis was performed without major hazard.

## 5 CONCLUSION

One‐stage hepaticojejunostomy could be a novel approach in selected patients (prior splenectomy with minimal and decompressed collaterals, prior hilar surgery and a thin‐built patient), where no shuntable veins are available for prior shunt surgery, provided all precautions are taken before and at the time of surgery by an experienced team.

## CONFLICT OF INTEREST

The authors declare that they have no competing interests.

Consent for Publication: Written informed consent was obtained from the participants for publication of this article and accompanying images.

## AUTHORS CONTRIBUTION

NP: collected the data, wrote the paper, and made the final revision. LA: collected data. LSJ and SA: coordinated the study and language revision. All authors: read and approved the final manuscript.
